# Impact of Decreased Transmural Conduction Velocity on the Function of the Human Left Ventricle: A Simulation Study

**DOI:** 10.1155/2020/2867865

**Published:** 2020-04-03

**Authors:** Jiří Vaverka, Jiří Moudr, Petr Lokaj, Jiří Burša, Michal Pásek

**Affiliations:** ^1^Institute of Solid Mechanics, Mechatronics and Biomechanics, Faculty of Mechanical Engineering, Brno University of Technology, Brno, Czech Republic; ^2^Department of Physiology, Faculty of Medicine, Masaryk University, Brno, Czech Republic; ^3^Department of Internal Medicine and Cardiology, University Hospital Brno, Brno, Czech Republic; ^4^Institute of Thermomechanics, Czech Academy of Science, Prague, Czech Republic

## Abstract

This study investigates the impact of reduced transmural conduction velocity (TCV) on output parameters of the human heart. In a healthy heart, the TCV contributes to synchronization of the onset of contraction in individual layers of the left ventricle (LV). However, it is unclear whether the clinically observed decrease of TCV contributes significantly to a reduction of LV contractility. The applied three-dimensional finite element model of isovolumic contraction of the human LV incorporates transmural gradients in electromechanical delay and myocyte shortening velocity and evaluates the impact of TCV reduction on pressure rise (namely, (*dP*/*dt*)_max_) and on isovolumic contraction duration (IVCD) in a healthy LV. The model outputs are further exploited in the lumped “Windkessel” model of the human cardiovascular system (based on electrohydrodynamic analogy of respective differential equations) to simulate the impact of changes of (*dP*/*dt*)_max_ and IVCD on chosen systemic parameters (ejection fraction, LV power, cardiac output, and blood pressure). The simulations have shown that a 50% decrease in TCV prolongs substantially the isovolumic contraction, decelerates slightly the LV pressure rise, increases the LV energy consumption, and reduces the LV power. These negative effects increase progressively with further reduction of TCV. In conclusion, these results suggest that the pumping efficacy of the human LV decreases with lower TCV due to a higher energy consumption and lower LV power. Although the changes induced by the clinically relevant reduction of TCV are not critical for a healthy heart, they may represent an important factor limiting the heart function under disease conditions.

## 1. Introduction

Cardiac conduction velocity (CV), the speed with which an electrical impulse propagates through the cardiac tissue, is one of the most important electrophysiological characteristics of heart muscle. In comparison with normal hearts, the myocardial CV was found to be significantly reduced in diseased animal and human hearts [1–4]. The reduction of CV was shown to increase the risk of reentrant activities that can lead to cardiac arrhythmias (for review, see King et al. [5]).

In human cardiac muscle, the CV consists of two components, the longitudinal (between 60 and 70 cm/s [4]) and the transversal (TCV, around 50 cm/s [4]). The transmural decrease of electromechanical delay (EMD) from endocardium to epicardium (EMD gradient ~2.1 ms/mm [6]) helps, in combination with TCV, to synchronize the onset of contraction in individual layers of the left ventricle (LV) [6]. However, there are, to our best knowledge, no published experimental results on the impact of TCV reduction on the ventricle contractility. Thus, it is unclear whether the clinically observed decrease of TCV and the corresponding transmural desynchronization of LV contraction contributes to a reduction of LV contractility or whether it rather represents a consequence of pathological changes at a cellular level without any significant effect on the LV function.

An attempt to quantify the effect of CV reduction on mechanical response of the mammalian heart and basic hemodynamic parameters was undertaken recently by Yuniarti and Lim [7]. In their simulations using an integrated electromechanical model of the LV, the CV correlated with cardiac pumping efficacy. While a decrease of CV from 70 to 30 cm/s induced a relative reduction of ejection fraction (EF) and stroke work by ~7 and 12%, respectively, the ATP consumption increased by ~7%. However, the model was formulated for canine heart and did not incorporate transmural differences either in EMD or in myocyte shortening velocity (MSV) observed by Cordeiro et al. [8].

In the present study, our recently published three-dimensional finite element (FE) model of isovolumic contraction (IVC) of the human LV [6] incorporating transmural gradients in EMD and MSV was used to examine the effect of changes in TCV on dynamics of LV pressure rise *dP*_V_/*dt* and IVC duration (IVCD) in a healthy human heart. In the second step, we used our lumped model of the human cardiovascular system to simulate the impact of the observed changes in (*dP*_V_/*dt*)_max_ and IVCD on cardiovascular hemodynamics and arterial pressure.

## 2. Methods

### 2.1. Model of the Human Left Ventricle

The impact of decrease in TCV on IVCD and (*dP*_V_/*dt*)_max_ was investigated using a three-dimensional FE model of the human LV created recently (in commercial FE software ANSYS®) to simulate the isovolumic phase of LV systole. The model is based on simplified ellipsoidal geometry meshed with hexahedral quadratic solid elements. Passive behaviour of myocardium (considered as purely elastic) was described with a transversely isotropic strain energy density function determining the constitutive relation between stresses and (elastic) strains. Active contraction of myocytes was modelled using special reinforcing elements with unidirectional stiffness which were created within the underlying solid mesh. Their active tension was generated using a simple approach based on fictitious thermal strains. By gradually decreasing a fictitious temperature of the reinforcing elements (with a certain coefficient of thermal expansion), negative thermal strains are developed and naturally counterbalanced by positive elastic strains; consequently, tension in the fibre direction is generated. In order to reflect the LV fibre architecture, the orientation of these elements was changed gradually across the wall between +60° and -60° (with respect to circumferential direction) on the endocardial and the epicardial surfaces, respectively [9]. Blood inside the LV cavity was modelled as incompressible liquid. In the control simulation, the electrical activation of LV myocardium was modelled under the assumption of simultaneous activation of the whole endocardial surface and subsequent endocardium-to-epicardium propagation at a constant TCV of 47 cm/s [1, 4]. Electrical activation time for each element was calculated as a ratio of the distance of the element from the endocardial surface and of the TCV value (under control conditions). The same calculation was applied with decreased TCV in the simulations of pathological conditions. Transmurally heterogeneous values of EMD and MSV were prescribed in all simulations following Cordeiro et al. [8]. As the contractile elements generate tension, the intraventricular pressure rises until the systemic diastolic blood pressure (80 mmHg) is reached. By decreasing the TCV (while keeping the other parameters unchanged), different time-pressure curves were calculated for various levels of myocardial conductivity. (*dP*_V_/*dt*)_max_ and IVCD were evaluated from these curves for each case. Besides the pressure data, wall stress in the direction of fibres was assessed, as well as the total strain energy accumulated in the LV walls in the end of the IVC (*SE*_IVC_) which reflects its energetic demands. For further details regarding the FE model and simulation conditions, the reader is referred to our previous paper [6].

The basic exploration of the impact of decrease in TCV on IVCD and (*dP*_V_/*dt*)_max_ was done by comparing the model outputs in control conditions and under TCV reduced to 50% (as observed by Taggart et al. [4] in patients after 3 minutes of ischemia).

### 2.2. Model of the Cardiovascular System

**Figure 1:**
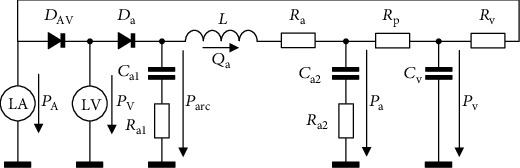
Electrical equivalent scheme of the model of left heart and systemic circulation. The individual symbols in the scheme stand for the left atrium and ventricle (LA, LV); atrioventricular and aortic valves (*D*_AV_, *D*_a_); inertia of blood (*L*); resistance against the blood flow in aorta, in peripheral vessels, and in the terminal part of the venous system (*R*_a_, *R*_p_, *R*_v_); viscoelastic compliance of the initial segment of aortic arch (*C*_a1_, *R*_a1_) and of the remaining aorta (*C*_a2_, *R*_a2_); and elastic compliance of the terminal part of the venous system (*C*_v_). The symbols *P*_A_, *P*_V_, *P*_arc_, *P*_a_, and *P*_v_ stand for the pressures in the left atrium, left ventricle, aortic arch, aorta, and venous system. *Q*_a_ represents blood flow in aorta. The values of individual parameters are specified in Table 1.

To simulate the impact of the observed changes in (*dP*_V_/*dt*)_max_ and IVCD on cardiovascular hemodynamics and arterial pressure, we reduced and modified our previously developed Windkessel (WK) model [10] describing the interaction of the heart with the vascular system. The reduced version of the WK model incorporates only the functions of the LV and left atrium (LA) that are necessary for the simulation of effects investigated in this study. The electrical equivalent scheme of the model is illustrated in Figure 1. In this model, the function of atrioventricular and aortic valves is represented by the marks of diodes (*D*_AV_, *D*_a_) with intrinsic resistances (*R*_DAV_, *R*_Da_) and the resistance of vessels against blood flow by the marks of a resistor (*R*_a_, *R*_p_, *R*_v_). Distensibility of the individual types of vessels (their viscoelastic compliance [11]) is represented by the marks of a capacitor (*C*_a1_, *C*_a2_, *C*_v_) in combination with resistors (*R*_a1_, *R*_a2_), and the inertia of blood is symbolised by the inductor (*L*). The volume of blood pumped repeatedly by the LV into the arterial system creates characteristic changes of arterial pressure known as pulse waves. Propagation of these waves along arteries and the pressure gradient between arterial and venous system underlay the blood circulation. The function of the LV is based on two important mechanisms influencing the time course of blood pressure development, the Frank–Starling mechanism, and the law of Laplace. Thus, the model involves all key events affecting systemic blood circulation and represents a more elaborated system than those published previously (see reviews by Zhou et al. [12] and Westerhof et al. [13]).

#### 2.2.1. Implementation of the Frank–Starling Mechanism

**Figure 2:**
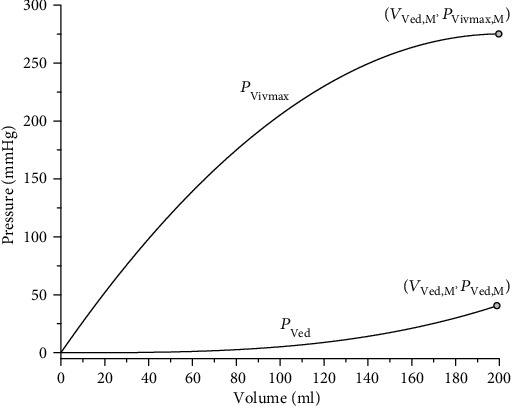
Pressure-volume diagram showing the passive end-diastolic pressure-volume curve (*P*_Ved_ versus *V*_Ved_) and isovolumic maxima curve (*P*_Vivmax_ versus *V*_Ved_) formulated in the WK model to reflect values in the human LV [14]. The points (*V*_Ved,M_, *P*_Ved,M_) and (*V*_Ved,M_, *P*_Vivmax,M_) represent values at theoretically maximal diastolic filling.

The Frank–Starling mechanism defines the relation between end-diastolic volume (*V*_Ved_) and strength of cardiac muscle contraction; it was implemented into the model by means of the following 3^rd^ and 2^nd^ order polynomial equations:

(1) $$\begin{array}{c} P_{\text{Ved}}=a_{\text{V}}V_{\text{Ved}}^{3}, \end{array}$$

(2) $$\begin{array}{c} P_{\text{Vivmax}}=P_{\text{Vivmax},\text{M}}-b_{\text{V}}{\left(V_{\text{Ved}}-V_{\text{Ved},\text{M}}\right)}^{2}, \end{array}$$

where *P*_Ved_ and *P*_Vivmax_, respectively, stand for end-diastolic ventricular pressure and the isovolumic maximum ventricular pressure (that could be achieved during persisting IVC at a given end-diastolic volume *V*_Ved_), and *P*_Vivmax,M_ represents the peak value of *P*_Vivmax_ (275 mmHg) achievable at *V*_Ved_ of 200 ml (*V*_Ved,M_). The related points (*V*_Ved,M_, *P*_Ved,M_) and (*V*_Ved,M_, *P*_Vivmax,M_) (see Figure 2) were then used to compute parameters *a*_V_ and *b*_V_ from the relations:

(3) $$\begin{array}{c} a_{\text{V}}=\frac{P_{\text{Ved},\text{M}}}{V_{\text{Ved},\text{M}}^{3}},\\ b_{\text{V}}=\frac{P_{\text{Vivmax},\text{M}}}{V_{\text{Ved},\text{M}}^{2}}. \end{array}$$

#### 2.2.2. Implementation of the Law of Laplace

LV is simplified in this model to a spherical shape with inner radius *r* and wall thickness *h*. Consistently with the law of Laplace, the internal pressure *P*_V_ induced by normal stress *σ*_V_ in the wall of the model (formulated below) was computed as

(4) $$\begin{array}{c} P_{\text{V}}=\sigma_{\text{V}}A_{\text{V}}, \end{array}$$

where, from the condition of force equilibration, it follows that

(5) $$\begin{array}{c} A_{\text{V}}=\frac{2h}{r}+{\left(\frac{h}{r}\right)}^{2}. \end{array}$$

The approximation of the LV by a sphere allows us to express the volume of LV cavity as

(6) $$\begin{array}{c} V_{\text{V}}=\frac{4}{3}\pi r^{3}, \end{array}$$

and the volume of LV wall (heart muscle) as

(7) $$\begin{array}{c} V_{\text{m}}=\frac{4}{3}\pi {\left(r+h\right)}^{3}-\frac{4}{3}\pi r^{3}=\frac{4}{3}\pi \left(3r^{2}h+3{rh}^{2}+h^{3}\right). \end{array}$$

By combining equations (6) and (7), we obtain a cubic equation:

(8) $$\begin{array}{c} \frac{V_{\text{m}}}{V_{\text{V}}}=3\frac{h}{r}+3{\left(\frac{h}{r}\right)}^{2}+{\left(\frac{h}{r}\right)}^{3}. \end{array}$$

The real root *h*/*r* in equation (8) can be then expressed as

(9) $$\begin{array}{c} \frac{h}{r}={\left(\frac{V_{\text{m}}}{V_{\text{V}}}+1\right)}^{1/3}-1, \end{array}$$

which allows us to formulate *A*_V_ as a function of *V*_m_ and *V*_V_ in the form

(10) $$\begin{array}{c} A_{\text{V}}=2\left[{\left(\frac{V_{\text{m}}}{V_{\text{V}}}+1\right)}^{1/3}-1\right]+{\left[{\left(\frac{V_{\text{m}}}{V_{\text{V}}}+1\right)}^{1/3}-1\right]}^{2}. \end{array}$$

As *V*_m_ is constant during the whole heart cycle (muscles consist of 95% of incompressible water) and *V*_V_ decreases after the opening of the aortic valve, the increase of *A*_V_ resulting from equation (10) contributes to the rise of *P*_V_ during the ejection phase.

#### 2.2.3. Implementation of Muscle Contraction and Relaxation

For the mathematical formulation of the muscle contraction and relaxation during one cardiac cycle, we used the following function in the model:

(11) $$\begin{array}{c} f_{\text{Vc}}=\exp\left\{-{\left[\text{abs}\left(t-t_{\text{Vmax}}\right)k_{\text{V}1}\right]}^{i_{\text{V}1}}-{\left[\text{abs}\left(t-t_{\text{Vmax}}\right)k_{\text{V}2}\right]}^{i_{\text{V}2}}\right\}, \end{array}$$

where constants *k*_V1_ and *k*_V2_ and exponents *i*_V1_ and *i*_V2_ control the contraction/relaxation rate and *t*_Vmax_ is the time from the origin of the excitation (in the sinoatrial node) to the maximal contraction of LV. The development of stress *σ*_V_ in the ventricle wall during the cardiac cycle was described by the following equation:

(12) $$\begin{array}{c} \sigma_{\text{V}}=\frac{a_{\text{V}}V_{\text{V}}^{3}}{A_{\text{V}}}+\frac{P_{\text{Vivmax},\text{M}}-b_{\text{V}}{\left(V_{\text{Ved}}-V_{\text{Ved},\text{M}}\right)}^{2}-a_{\text{V}}V_{\text{V}}^{3}}{A_{\text{V}}}f_{\text{Vc}}K_{\text{Vc}}f_{\text{Ve}}, \end{array}$$

where *K*_Vc_ represents a coefficient of contractility (1 in control conditions) which reflects the level of neural activity and fitness of the heart and *f*_Ve_ is a function that reduces *σ*_V_ during the ejection along with the decrease of *V*_V_ and, hence, stretch of muscle fibres. This function was formulated to ensure the physiological time course of *P*_V_ [15] and values of diastolic and systolic arterial pressures during a steady cardiac cycle under control conditions [16]. Its mathematical form is

(13) $$\begin{array}{c} f_{\text{Ve}}=1-\frac{1}{K_{\text{e}}}{\left[-\ln\left(\frac{V_{\text{V}}}{V_{\text{Ved}}}\right)\right]}^{i_{\text{e}}}, \end{array}$$

and numerical values of parameters *K*_e_ and *i*_e_ are specified in Table 1.

An analogical approach as used for the formulation of LV function was applied to describe the function of LA. However, because at rest the contribution of LA to the performance of normal left heart is small [17], the description of LA was simplified. The relation between LA pressure (*P*_A_) and volume (*V*_A_) during the LA filling was formulated by means of 5^th^ order polynomial equation:

(14) $$\begin{array}{c} P_{\text{A}}=a_{\text{A}}V_{\text{A}}^{5}, \end{array}$$

where

(15) $$\begin{array}{c} a_{\text{A}}=\frac{P_{\text{A},\text{M}}}{V_{\text{A},\text{M}}^{5}},\\ V_{\text{A},\text{M}}=100\text{ ml},\\ P_{\text{A},\text{M}}=30\text{ mmHg}. \end{array}$$

The development of *P*_A_ during the whole cardiac cycle was then described by the equation:

(16) $$\begin{array}{c} P_{\text{A}}=a_{\text{A}}V_{\text{A}}^{5}+f_{\text{A}c}\left[7.5-b_{\text{A}}{\left(V_{\text{A}}-V_{\text{A},\text{M}}\right)}^{2}\right], \end{array}$$

where *b*_A_ = 0.00075 mmHg/ml^2^ and *f*_Ac_ represent LA contraction defined by the term:

(17) $$\begin{array}{c} f_{\text{Ac}}=\exp\left\{-{\left[abs\left(t-t_{\text{Amax}}\right)k_{\text{A}}\right]}^{i_{A}}\right\}. \end{array}$$

Here, constant *k*_A_ and exponent *i*_A_ control the contraction/relaxation rate of LA and *t*_Amax_ is the time from the origin of the excitation (in the sinoatrial node) to the maximal contraction of LA.

The parameters of the WK model (see Table 1) were recursively optimised by the least square method using normalised differences between the model outputs and the required values (see Table 2—standard) to make the model capable to mimic the physiological properties of the human cardiovascular system. The core of the model consisting from 6 differential and two algebraic equations is presented in the appendix. The model was implemented in the computational system MATLAB14A-Simulink (MathWorks, Inc.). The numerical computation of the system of differential equations was performed using solver ODE-45 (with absolute and relative errors set to 10^−4^ and 5·10^−6^, respectively). To obtain steady cycles under control conditions or decreased TCV, the model was run for 60 s of equivalent real time; prolongation of the simulation time to 120 s did not change the model output values by more than 0.01%. The stability of the model was tested by running the model at parameters changed by 30 and 50%, specifically those related to cardiac contractility (*K*_vc_), physical properties of the valves (*R*_DAV__,_*R*_Da_), and of the vessels (*R*_a1_, *R*_a2_, *R*_a_, *R*_p_, *R*_v_, *C*_a1_, *C*_a2_, *C*_v_, *L*). In all the cases, the model converged and steady cycles were achieved within 60 s.

## 3. Results

### 3.1. Impact of Decreased Transmural Conduction Velocity on the Function of the Left Ventricle during Isovolumic Contraction

**Figure 3:**
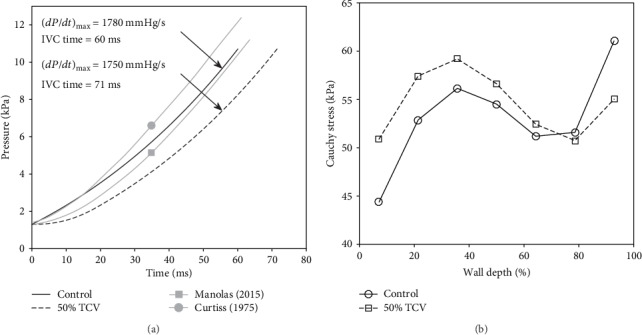
(a) Effect of a 50% decrease of TCV on pressure rise in the LV during IVC. Pressure development obtained in the control simulation is compared with two normal pressure traces (in grey) digitized from literature [18, 19]. (b) Effect of a 50% decrease of TCV on distribution of stresses (in the direction of myofibres) across the LV wall (from endocardium—0% to epicardium—100%) in the end of IVC.

To explore the impact of decreased TCV on function of left ventricle during IVC, we used our FE model of LV and simulated the development of intraventricular pressure and underlying changes in wall stress under control conditions, and when TCV was slowed by 50% (see methods for detailed explanation). The results illustrated in Figure 3(a) show that such decrease in TCV would cause an increase of IVCD from 60 to 71 ms and a slight reduction of (*dP*_V_/*dt*)_max_ from 1780 to 1750 mmHg/s. For comparison, Figure 3(a) includes also two clinically measured normal pressure traces (digitized from literature [18, 19]) which demonstrate a good agreement between our FE model and clinical observations. Besides the changes in values of the parameters derived from the pressure traces, an increased wall stress was detected in the endocardial and midmyocardial layers of the LV at the end of IVC (Figure 3(b)). This elevation of wall stress was reflected by an increase of *SE*_IVC_ from 441 to 466 mJ (by 6%) indicating higher energetic demands of IVC when TCV was slowed.

### 3.2. Impact of Decreased Transmural Conduction Velocity on Left Ventricular Performance and Blood Pressure

**Figure 4:**
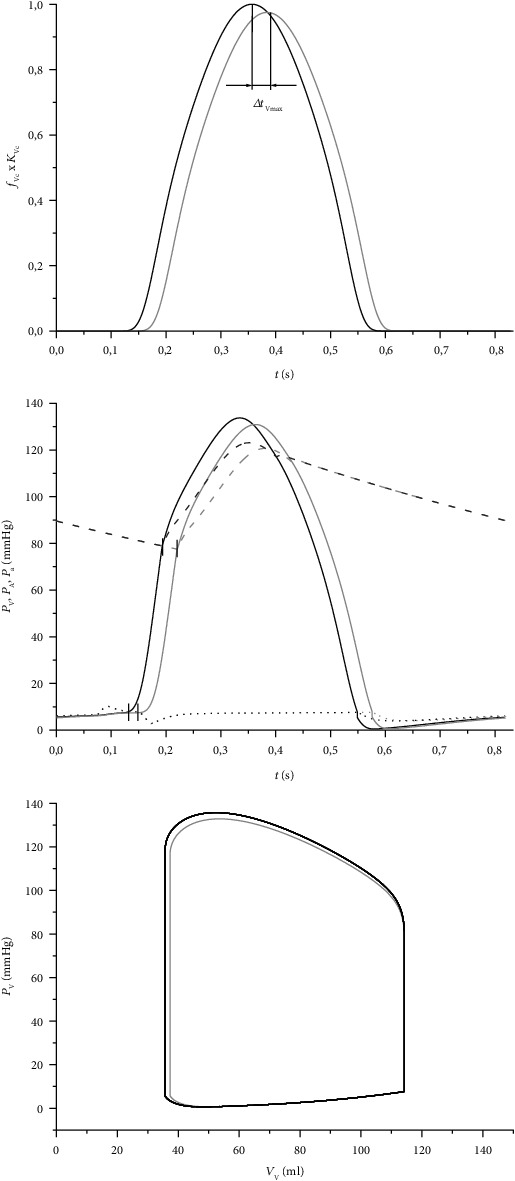
Simulation of function of systemic cardiovascular circuit during a steady cycle at resting heart rate (72 beats/min, stimulation interval 0.8333 s) in control conditions (black lines) and after incorporation of changes (increase of *t*_Vmax_ and decrease of *K*_Vc_) resulting in prolongation of IVCD and reduction of (*dP*_V_/*dt*)_max_ (grey lines); these changes were obtained by means of the FE model after decrease of TCV to 50%. The traces in the upper graph represent the time courses of LV contraction, and Δ*t*_Vmax_ represents a delay of the maximum contraction under the reduced TCV against control conditions. The middle graph shows the time course of development of *P*_V_ (solid), *P*_A_ (dotted), and *P*_a_ (dashed) under both explored conditions; the small black vertical lines mark the beginning and end of IVC. The bottom graph shows the corresponding *P*_V_–*V*_V_ diagrams with their loop area representing the LV stroke work; for comparison with *P*_V_–*V*_V_ diagram measured in normal human LV see Figure 12.2 in [20].

The analysis described in the previous section indicates that 50% reduction of TCV causes a significant prolongation of IVCD by 18% and a small reduction of (*dP*_V_/*dt*)_max_ by 2%. To incorporate this effect into the WK model, we increased the *t*_Vmax_ to 0.3885 and reduced *K*_Vc_ to 0.982. Such change consistently resulted in the increase of IVCD (from 60 to 71 ms) and reduction of (*dP*_V_/*dt*)_max_ from 1783 to 1750 mmHg/s during the first cycle. The consequences of these changes on cardiovascular hemodynamics and arterial pressure in a steady cycle (after 60 s of 1.2 Hz stimulation) are illustrated in Figure 4. The simulations reveal that these changes implicate a delayed and weakened contraction (upper graph), with consequences for time distribution and magnitude of LV and arterial pressures (middle graph), and for LV power (*W*_LV_) computed from the area of the loop in the *P*_V_–*V*_V_ diagram (bottom graph). The quantitative analysis of this effect summarized in Table 2 shows a reduction of EF, cardiac output (CO), and *W*_LV_ by ~2, 2, and 4%, respectively, and the consequent decrease of the systolic and diastolic arterial pressures (*P*_a,s_ and *P*_a,d_) by 2 and 1%, respectively.

**Figure 5:**
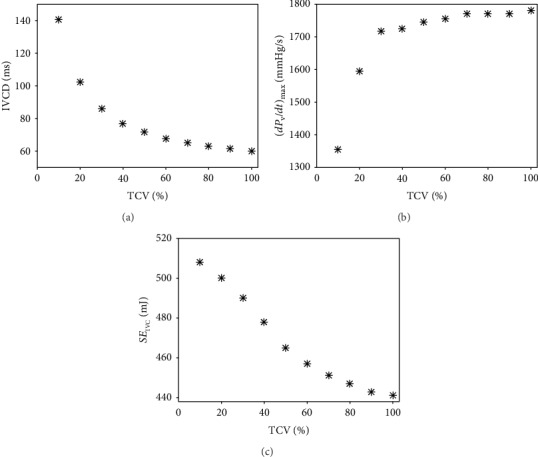
Impact of TCV reduction on three indexes characterizing left ventricle contractility and energetic demands of IVC in the model: (a) IVCD, (b) (*dP*_V_/*dt*)_max_, and (c) strain energy. 100% TCV represents a normal human LV.

To assess the instantaneous impact of TCV reduction on IVCD, (*dP*_V_/*dt*)_max_, and *SE*_IVC_ in greater detail, we repeated the simulations with the FE model using TCV values between 100 and 10%. The results presented in Figure 5 show that a reduction of TCV from 100 to 50% caused a nearly linear increase of IVCD and decrease of (*dP*_V_/*dt*)_max_. However, further reduction of TCV below 50% caused a highly nonlinear change of both of these contractility indexes. On the other hand, the strain energy exhibited approximately linear dependence on TCV in the whole range of the explored values. Adjusting the WK model for values of IVCD and (*dP*_V_/*dt*)_max_ that were obtained by the FE model at TCV reduced to 30, 20, and 10% of the control value resulted in a reduction of *W*_LV_ and CO, respectively, by ~7, 20, and 41% and by ~4, 10, and 23%. Consequently, the *P*_a,s_ and *P*_a,d_ decreased, respectively, by ~4, 10, and 22% and by ~3, 9, and 20% giving values *P*_a,s_/*P*_a,d_ of 120/78, 113/73, and 98/64.

To sum up, these simulations suggest that the isolated impact of TCV on LV performance is rather small when TCV is reduced from 100 to 50% of its control value but that it increases progressively under further reduction of TCV.

## 4. Discussion

Cardiac CV is a parameter determining the velocity of depolarization wave propagation through the myocardium. As the excitation is rapidly distributed to the whole inner endocardial layer by the cardiac conduction system and extensive net of Purkinje fibres in human LV [22], the critical factor responsible for the propagation of excitation through the ventricular wall is TCV. Although the CV and the related TCV have been observed to decrease in diseased human hearts [1, 2, 4], it is not clear how much this decrease contributes to the reduction of LV contractility. To answer this question, we used our previously published FE model of human LV and performed simulations showing the effect of slowed TCV on (*dP*_V_/*dt*)_max_ and IVCD. Subsequently, the impact of the changes of (*dP*_V_/*dt*)_max_ and IVCD—induced in the FE model by the lower TCV—on the cardiovascular hemodynamics and the arterial pressure was simulated using a modified version of our lumped model of systemic cardiovascular circuit.

### 4.1. Causes of Slowed Transmural Conduction Velocity in the Cardiac Left Ventricle

In principle, the TCV is determined by the rate of local depolarisation of cardiomyocytes and by the rate of excitation propagation between them (in transversal direction). These two determinants of TCV are closely related to the amplitude of fast Na^+^ current (*I*_Na_) in ventricular myocytes, to their membrane capacitance (*C*_m_), and to their transversal resistance (*R*_t_) controlled by cardiac gap junctions (mainly formed by connexin43, Cx43) which realize the cell-to-cell couplings. Changes of these three factors underlying slowed TCV have been observed in a variety of pathophysiological conditions. Firstly, *I*_Na_ is reduced by the impaired function of Na^+^ channels that arise clinically during heart failure, ischemia, tachycardia, or as a consequence of treatment with class I antiarrhythmic drugs [5]. Such reduction may be also induced by Na^+^ channel mutations that occur in Lenègre disease, Brugada syndrome, sick sinus syndrome, and atrial fibrillation [5, 23]. Secondly, *C*_m_ is usually substantially increased under ventricular hypertrophy which reflects the thickening and elongation of ventricular myocytes. This change is known to be induced by hypertension [24], valvular disease (mitral valve regurgitation or aortic valve stenosis [25, 26]), congenital heart disease (such as patent ductus arteriosus or coarctation of the aorta [27, 28]), and a primary disease of the myocardium which directly cause hypertrophy (hypertrophic cardiomyopathy [29]). Finally, *R*_t_ may increase due to gap junction decoupling following ischemia, fibrotic change of the heart tissue (e.g., after myocardial infarction) [30], or as a result of mutations of genes encoding gap junction protein connexions [31]. Besides, downregulation and dephosphorylation of Cx43 have been reported to contribute to an increase of *R*_t_ and thus to a slower propagation of excitation through the LV wall in failing hearts [1, 3].

### 4.2. Effect of Slowed Transmural Conduction Velocity on the Function of the Cardiovascular System

The simulations on the FE model suggest that the isolated reduction of TCV results in a prolongation of IVCD and decrease of (*dP*_V_/*dt*)_max_. To explore the effect of these two changes on the LV performance and cardiovascular hemodynamic, the modified form of our WK model [10] was used (see Figure 1). An analysis of the WK model parameters showed that the above effect observed in the FE model could be replicated most effectively by an increase of *t*_Vmax_ which controls the time of activation of LV contraction (equation (11)), and by a decrease of coefficient *K*_Vc_ which determines the strength of cardiac muscle contraction (equation (12)). Implementation of the effects of 50% reduction of TCV (i.e., ~18% increase of IVCD and ~2% decrease of (*dP*_V_/*dt*)_max_, see Figure 3(a)) in the WK model affected its behaviour only moderately: EF and CO decreased by 2%, *W*_LV_ by 4%, and the effect on *P*_a,s_ and *P*_a,d_ was small. However, it is important to emphasize that in fact the evaluated impacts on both *SE*_IVC_ and *W*_LV_ sum up. While the FE model shows an increase of *SE*_IVC_ by 6%, the WK model shows a decrease of *W*_LV_ by 4% under these conditions. It means that during contraction, the LV consumes more energy to develop wall stress but its contractile power declines. Consequently, the resulting efficiency of the heart contraction decreases approximately by 10%; clearly, this is only true when the increase of energy consumption during the ejection phase (not included in the FE model) is proportional to that during IVC. In any case, such a decrease of efficiency of heart contraction may be significant for the efficiency of blood supply, especially in combination with some other pathologies impairing the LV function. The simulations also predict that the above described effects of lower TCV would increase substantially if TCV dropped under 50%. The reason of this increase was a continuous rise of *SE*_IVC_ (Figure 5) and a progressive reduction of *W*_LV_ (see Section 3.2). Hence, the reduction of TCV to 30, 20, and 10% of control value resulted in a decrease of contraction efficacy of LV by 16, 29, and nearly 50%, respectively.

The described effects are fully consistent with the recent work by Yuniarti and Lim [7] presenting simulations based on an electromechanical model of canine heart coupled with a lumped model of circulatory system. Their results also showed an increase in the electrical activation time (equivalent to IVCD) and in end-systolic volume with reduction of the CV, while systolic pressure, stroke volume, and stroke work decreased rather moderately. All these tendencies correspond to those depicted in Figures 3 and 4 and suggest that clinically relevant reduction of TCV does not affect critically the function of the cardiovascular system under normal conditions.

### 4.3. Clinical Implications

The reduction of CV is usually mirrored by the increased duration of QRS complex in ECG records. QRS prolongation (>120 ms) is a significant predictor of LV systolic dysfunction in patients with heart failure [32] and is known to be accompanied by higher propensity to arrhythmias [33, 34]. On the other hand, because QRS can be affected by disorders of cardiac electrical conduction system (e.g., by left bundle branch block), the prolonged QRS does not necessarily mean that intraventricular CV is slowed down. To unambiguously differentiate between the causes of QRS prolongation, new diagnostic methods allowing to monitor LV activation pattern [35, 36] would be very helpful in clinical practice. The possibility to directly identify a reduced TCV and knowledge of its relation to cardiac contraction efficiency might be an impulse for the development of new and more effective therapies targeted to normalization of intraventricular spread of excitation in patients with cardiac disease. Besides reperfusion of heart tissue, this could involve also a potentiation or upregulation of some membrane transporters (e.g., sodium channels or gap junction channels) which could lead to normalization of cellular excitability and intercellular electrical conductance. A future more elaborated version of the model incorporating cell-to-cell electrical interaction could be also helpful for mapping of arrhythmogenic substrate in the myocardium in patients with a hereditary cardiac disease such as Brugada syndrome.

### 4.4. Limitations of the Model

The FE model used in this study is based on an idealized (ellipsoidal) geometry of the LV. It also employs a simplified electrical activation pattern taking into consideration the propagation of the electrical signal only in the transmural direction; consequently, the entire endocardium is activated simultaneously. Nevertheless, assuming that propagation of depolarisation around the LV cavity is much faster than in the transmural direction [37], this represents a reasonable approximation. Also, the passive mechanical behaviour of human myocardium is orthotropic [38] rather than transversely isotropic as applied in our model; thus, further improvement could be achieved by employing an orthotropic hyperelastic model, e.g., that proposed by Holzapfel and Ogden [39]. Finally, besides a dramatic transmural variation, moderate changes in fibre direction have been observed also in circumferential direction and between base and apex [9]. These minor variations are not included in our model. We believe the mentioned limitations may change the results quantitatively but without a significant impact on the drawn conclusions.

## 5. Conclusions

On the basis of combination of two computational models, FE model of left ventricle and WK model of cardiovascular hemodynamics, the presented study suggests that the pumping efficacy of human heart decreases with lower TCV due to a higher energy consumption and lower LV power. Although the observed changes induced by the clinically relevant reduction of TCV are not critical for healthy heart, they may represent an important factor limiting cardiac function when combined with other pathologies impairing contractility of the LV. As numerous heart pathologies are associated with TCV reduction, further exploration of the impact of TCV on the contractility of diseased hearts is needed.

## Figures and Tables

**Table 1:** Parameters of the WK model.

*R*_DAV_	0.012 mmHg·s/ml^∗^	*K*_Vc_	1
*R*_Da_	0.025 mmHg·s/ml^∗^	*k*_V1_	5.68722
*R*_a1_	0.05 mmHg·s/ml	*k*_V2_	5.2270
*R*_a2_	0.026 mmHg·s/ml	*i*_V1_	2.0224
*R*_a_	0.0001 mmHg·s/ml	*i*_V2_	9.11538
*R*_p_	1 mmHg·s/ml	*t*_Vmax_	0.3568 s
*R*_v_	0.01 mmHg·s/ml	*K*_e_	1.355
*C*_a1_	0.08 ml/mmHg	*i*_e_	0.35
*C*_a2_	1.3 ml/mmHg	*k*_A_	24
*C*_v_	70 ml/mmHg	*i*_A_	7
*L*	0.0003 mmHg·s^2^/ml	*t*_Amax_	0.12 s

^∗^Valid only for open state. Under closed state (when *P*_V_ > *P*_A_ or *P*_arc_ > *P*_V_), the corresponding resistance (*R*_DAV_ or *R*_Da__)_ is set to 10^4^ mmHg·s/ml.

**Table 2:** Parameters representing cardiovascular hemodynamics and LV performance in a steady cycle obtained from literature (Standard), from the model under control conditions (Control), and at TCV decreased to 50% (50% TCV).

	Standard	Control	50% TCV
*P*_a,s_	120 mmHg	125 mmHg	122 mmHg
*P*_a,d_	80 mmHg	80 mmHg	79 mmHg
(*dP*_V_/*dt*)_max_	1780 mmHg/s	1783 mmHg/s	1751 mmHg/s
*V*_V,ed_	120 ml	114 ml	114 ml
*V*_V,es_	40 ml	36 ml	37 ml
IVCD	60 ml	60 ms	71 ms
EPD	210 ml	211 ms	213 ms
EF	67%	69%	67%
CO	5600 ml/min	5653 ml/min	5538 ml/min
*W*_LV_	1.5 W	1.51 W	1.45 W

*P*_a,s_: systolic pressure in the aorta; *P*_a,d_: diastolic pressure in the aorta; *V*_V,ed_: end-diastolic volume in the LV; *V*_V,es_: end-systolic volume in the LV; EPD: duration of ejection phase; EF: ejection fraction; CO: cardiac output; *W*_LV_: power of the LV. The standard values of parameters were taken from [6, 21].

## Data Availability

The datasets generated and analysed during the current study are available from the corresponding author upon request.

## Appendix

### Differential Equations of the WK Model

Pressure induced by the elastic component of aortic arch

(A.1) $$\begin{array}{c} \frac{{dP}_{Ca1}}{dt}=\frac{\left(P_{\text{V}}-P_{\text{arc}}\right)/R_{\text{Da}}-Q_{\text{a}}}{C_{\text{a}1}}, \end{array}$$

where

(A.2) $$\begin{array}{c} P_{\text{arc}}=P_{\text{V}}\frac{R_{\text{a}1}}{R_{\text{Da}}+R_{\text{a}1}}+P_{\text{Ca}1}\frac{R_{\text{Da}}}{R_{\text{Da}}+R_{\text{a}1}}-Q_{\text{a}}\frac{R_{\text{a}1}R_{\text{Da}}}{R_{\text{Da}}+R_{\text{a}1}}. \end{array}$$

Pressure induced by the elastic component of the aorta

(A.3) $$\begin{array}{c} \frac{{dP}_{Ca2}}{dt}=\frac{Q_{\text{a}}-\left(P_{\text{a}}-P_{\text{v}}\right)/R_{\text{p}}}{C_{a2}}, \end{array}$$

where

(A.4) $$\begin{array}{c} P_{\text{a}}=P_{\text{v}}\frac{R_{\text{a}2}}{R_{\text{p}}+R_{\text{a}2}}+P_{\text{Ca}2}\frac{R_{\text{p}}}{R_{\text{p}}+R_{\text{a}2}}+Q_{\text{a}}\frac{R_{\text{p}}R_{\text{a}2}}{R_{\text{p}}+R_{\text{a}2}}. \end{array}$$

Pressure induced by the elastic component of the venous system

(A.5) $$\begin{array}{c} \frac{{dP}_{\text{v}}}{dt}=\frac{\left(P_{\text{a}}-P_{\text{v}}\right)/R_{\text{p}}-\left(P_{\text{v}}-P_{\text{A}}\right)/R_{\text{v}}}{C_{\text{v}}}. \end{array}$$

Blood flow through the aorta

(A.6) $$\begin{array}{c} \frac{{dQ}_{\text{a}}}{dt}=\frac{\left(P_{\text{arc}}-P_{\text{a}}-R_{\text{a}}Q_{\text{a}}\right)}{L}. \end{array}$$

Volume of the left atrium

(A.7) $$\begin{array}{c} \frac{{dV}_{\text{A}}}{dt}=\frac{P_{\text{v}}-P_{\text{A}}}{R_{\text{v}}}-\frac{P_{\text{A}}-P_{\text{V}}}{R_{\text{DAV}}}. \end{array}$$

Volume of the left ventricle

(A.8) $$\begin{array}{c} \frac{{dV}_{\text{V}}}{dt}=\frac{P_{\text{A}}-P_{\text{V}}}{R_{\text{DAV}}}-\frac{P_{\text{V}}-P_{\text{arc}}}{R_{\text{Da}}}. \end{array}$$
